# A Mental Health and Well-Being Chatbot: User Event Log Analysis

**DOI:** 10.2196/43052

**Published:** 2023-07-06

**Authors:** Frederick Booth, Courtney Potts, Raymond Bond, Maurice Mulvenna, Catrine Kostenius, Indika Dhanapala, Alex Vakaloudis, Brian Cahill, Lauri Kuosmanen, Edel Ennis

**Affiliations:** 1 Department of Accounting, Finance & Economics Belfast United Kingdom; 2 School of Psychology Ulster University Coleraine United Kingdom; 3 School of Computing Ulster University Belfast United Kingdom; 4 Department of Health, Education and Technology Luleå University of Technology Luleå Sweden; 5 Nimbus Research Centre Munster Technological University Cork Ireland; 6 Department of Nursing Science University of Eastern Finland Kuopio Finland

**Keywords:** mental well-being, positive psychology, data analysis, health care, event log analysis, ecological momentary assessment, conversational user interface, user behavior, conversational agent, user interface, user data, digital health application, mental well-being, mobile health app, digital intervention

## Abstract

**Background:**

Conversational user interfaces, or chatbots, are becoming more popular in the realm of digital health and well-being. While many studies focus on measuring the cause or effect of a digital intervention on people’s health and well-being (outcomes), there is a need to understand how users really engage and use a digital intervention in the real world.

**Objective:**

In this study, we examine the user logs of a mental well-being chatbot called ChatPal, which is based on the concept of positive psychology. The aim of this research is to analyze the log data from the chatbot to provide insight into usage patterns, the different types of users using clustering, and associations between the usage of the app’s features.

**Methods:**

Log data from ChatPal was analyzed to explore usage. A number of user characteristics including user tenure, unique days, mood logs recorded, conversations accessed, and total number of interactions were used with k-means clustering to identify user archetypes. Association rule mining was used to explore links between conversations.

**Results:**

ChatPal log data revealed 579 individuals older than 18 years used the app with most users being female (n=387, 67%). User interactions peaked around breakfast, lunchtime, and early evening. Clustering revealed 3 groups including “abandoning users” (n=473), “sporadic users” (n=93), and “frequent transient users” (n=13). Each cluster had distinct usage characteristics, and the features were significantly different (*P*<.001) across each group. While all conversations within the chatbot were accessed at least once by users, the “treat yourself like a friend” conversation was the most popular, which was accessed by 29% (n=168) of users. However, only 11.7% (n=68) of users repeated this exercise more than once. Analysis of transitions between conversations revealed strong links between “treat yourself like a friend,” “soothing touch,” and “thoughts diary” among others. Association rule mining confirmed these 3 conversations as having the strongest linkages and suggested other associations between the co-use of chatbot features.

**Conclusions:**

This study has provided insight into the types of people using the ChatPal chatbot, patterns of use, and associations between the usage of the app’s features, which can be used to further develop the app by considering the features most accessed by users.

## Introduction

Chatbots, or conversational user interfaces can take diverse roles in supporting mental health. In particular, chatbots are becoming increasingly popular as digital mental health and well-being interventions, with initial evaluations of efficacy showing promise [1-3]. Chatbots may be targeted toward a variety of outcomes such as medication adherence, treatment compliance, aftercare support, delivery of appointment reminders, psychoeducation, user empowerment, and improvement in the self-management of mental health and well-being through monitoring mood or symptom change [1]. They can also be used to promote help-seeking [1]. The potential benefits are recognized by both practitioners and clients [2-5]. In addition to supporting those with mental ill health, digital technologies are also considered to have the potential for preventing mental health problems and for improving the overall mental health of the population [6].

Event logging plays an important role in modern IT systems with many apps logging their events to a local or remote server. These event logs can be used to determine the use of an app and identify user patterns. The analysis of user patterns involves event correlation—a conceptual interpretation procedure where new meaning is assigned to events that occur within a set time frame [7].

Event logging can be easily incorporated into digital products, including apps and websites. The most basic event log data consists of an anonymous unique identifier assigned to each individual, a date-time stamp, and an activity or event, but may also include other contextual variables. These user interaction log data provide useful information on how digital technologies are actually being used, providing valuable insights into user behavior [8]. Event log analysis typically focuses on quantitative data, but it may provide even greater insights when combined with qualitative data such as ecological momentary assessment (EMA) [8-11]. EMA involves asking questions, for example, “how do you feel right now,” repeatedly over a period of time in an individual’s own environment (ecology). Users answer EMA questions “in the moment,” which helps to avoid recall bias [11]. Previous trials with mental health chatbots have used app interaction data [12,13]. For example, participants trialing the mental health chatbot “Wysa” were characterized based on their app usage into more engaged users, termed “high users,” and less engaged users, or “low users” [13]. A multilingual mental health and well-being chatbot named “ChatPal” was developed to promote good mental well-being of citizens living in sparsely populated areas across Europe. Once the chatbot was released into the wild, all interactions between the chatbot and users were logged.

The aim of this research is to analyze event log data from the ChatPal chatbot with the objectives of providing insight into the different types of users using k-means clustering, exploring usage patterns, and associations between the usage of the app’s features.

## Methods

### Ethics Approval

This study received ethical approval from the Ulster University Research Ethics Committee (reference numbers REC.21.0021 and FCPSY-21-038-A), the Munster Technological University Research Ethics Committee (reference number MTU21034A), the Ethics Review Authority in Sweden (reference Etikprövningsmyndigheten number 2020-00808), and the University of Eastern Finland Committee on Research Ethics gave a supporting statement (statement 14/2021).

### Intervention

The ChatPal chatbot was co-designed with end users and developed as part of the ChatPal project [14], a collaboration between universities and mental health service providers. The remit of the project was to develop and evaluate a chatbot to promote the positive mental well-being of individuals living in rural areas across Europe. This was achieved using an iterative approach similar to a previous study on how users engage and are redirected through a chatbot for depression [15]. A prototype chatbot was released early to support individuals at the beginning of the COVID-19 pandemic, and feedback from this initial phase of the study was used to refine the chatbot [16]. This refined version was then trialed in Northern Ireland, the Republic of Ireland, Scotland, Sweden, and Finland. Individuals across these regions were recruited to take part in a pre-post intervention study, using the ChatPal app as they wished for a period of 12 weeks. The chatbot was also advertised on social media and was freely available on the Apple App Store and Google Play Store. The chatbot was developed based on the concept of positive psychology, with elements of psychological well-being and happiness. Known as the PERMAH model [17], it includes content to encourage positive emotions, engagement, relationships, meaning, accomplishment, and health. Based on mostly scripted conversations with predefined responses, ChatPal can maintain a basic dialogue with a user in order to advise them on how to maintain positive emotions and mental well-being. An overview of the content available in ChatPal can be found in Multimedia Appendix 1.

### Chatbot Development

**Figure 1:**
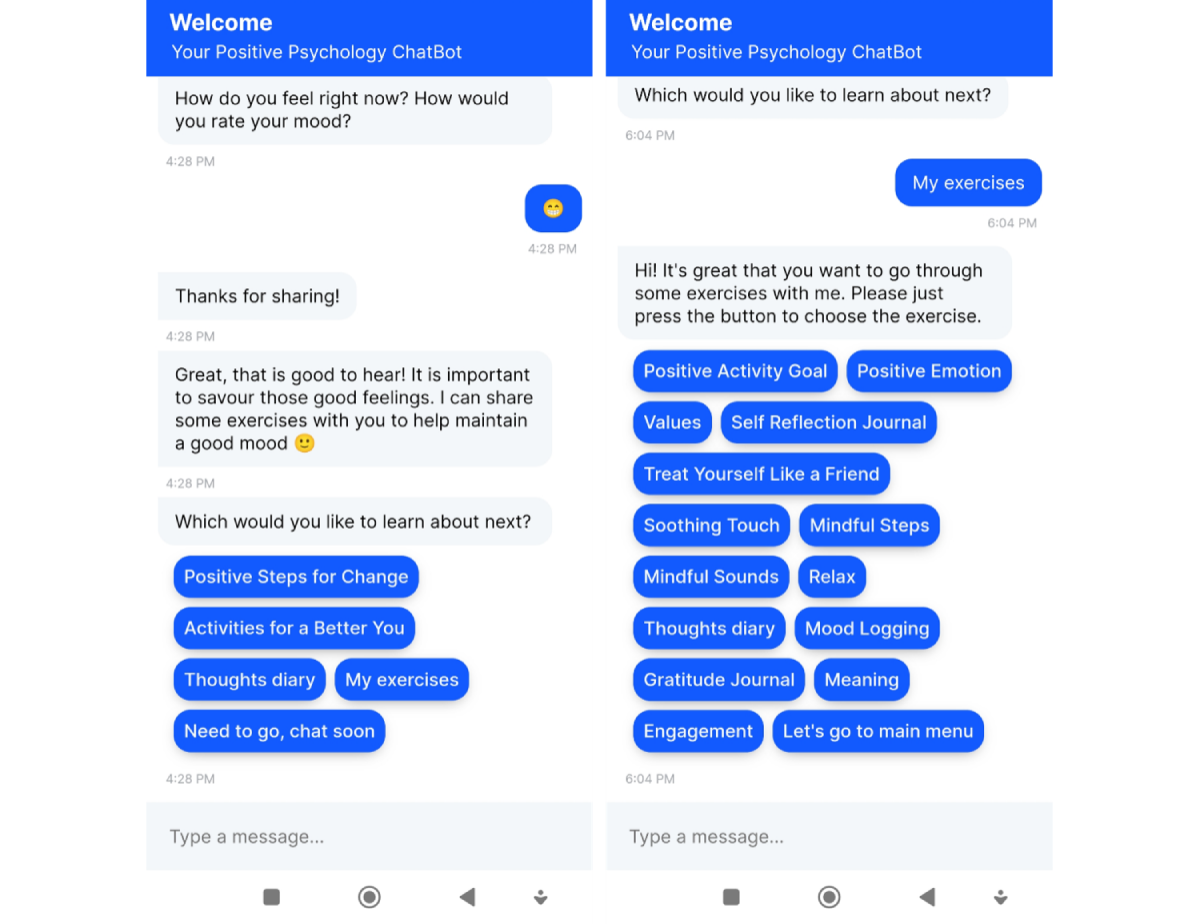
Screenshots of the ChatPal chatbot app.

The ChatPal chatbot was developed using the Rasa and PhoneGap frameworks. Rasa (Alan Nichol and Alex Weidauer) is an artificial intelligence (AI)–assisted framework for building contextual chatbots and provides the infrastructure and tools necessary for high-performing, resilient, proprietary contextual assistants. PhoneGap is an open-source framework for developing cross-platform mobile apps, including iPhone and Android. The PhoneGap framework was used to develop the front end of the ChatPal app, resulting in a cross-platform mobile app using HTML5, JavaScript, and CSS (Figure 1). Communication with the ChatPal backend is achieved using HTTP requests or responses. Upon receiving user inputs, the Rasa backend analyzes these inputs using its Natural Language Understanding unit, which extracts user intentions and relevant metadata from the input. Once the intentions and metadata are identified, corresponding actions and responses are decided by the AI Rasa core. ChatPal dialogues required additional functionality such as language selection, onboarding, log entries, and visualized responses (graphs), which were addressed by custom development in Rasa.

### User Log Data Provenance

The log data file initially contained all interactions between users and the app (including detailed app events) that occurred during a prepost study period (January 24-June 22, 2022). During data cleaning, it was necessary to identify only the events made by users and extract these user event log details for analysis. While users remained anonymous, each user was assigned a unique ID. Log entries were timestamped to facilitate the tracking of interactions over time. The app afforded users the opportunity to converse with the chatbot to help users manage their mental health, with their current mood being logged each time they used the chatbot. Users were also given the option to complete 5 questions relating to the World Health Organization-Five Well-being Index (WHO-5), a measure of current mental well-being [18].

### Data Analysis and Machine Learning

Jupyter notebooks [19] with the programming language Python were used to analyze the log data, with the matplotlib [20] and seaborn [21] libraries used to visualize the results. The scipy [22] and sklearn [23] libraries were used to normalize and perform k-means clustering, and the mlxtend [24] library was used for association rule mining.

**Figure 2:**
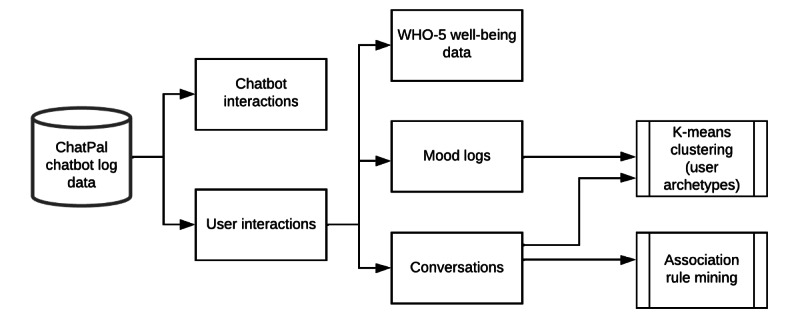
Methodology for log analysis. WHO-5: World Health Organization-5 well-being index.

Figure 2 shows the methodology for analyzing the ChatPal chatbot event log data. Data on each user were collated, including age, gender, and interactions with the chatbot. As only data pertaining to users older than 18 years was permitted, only users who had completed the age question could be included in the analysis. Of these users, those who did not complete the gender question were assigned an “unknown” status for gender.

### User Interactions

The total number of user interactions with the chatbot was examined across hours of the day to gain insight into daily patterns of use. The tenure (ie, the number of days from first to last use of the app) was calculated for each user along with the number of unique days of use. User retention was then calculated to discover the percentage of users still using the app over time.

### User Types

K-means clustering was used to determine the different types of users who interacted with the chatbot, with 6 features relating to the behavioral usage of the chatbot being identified within the log data (Table 1). Before submitting these features to the clustering algorithm, these features were normalized, resulting in the standardization of all variables to ranges between 0 and 1. In order to determine the optimum number of clusters, the k-means algorithm was applied to the data for clusters k=2 to k=15, with the average distance to the centroid from all data points (within-cluster sum of square) being calculated for each iteration. These were then plotted to identify the “pivot” where the resulting graph creates an “elbow,” which corresponds to the optimum k value (ie, the number of user groups or clusters that exist). The k-means algorithm was then used for this “k,” and the resulting output was visualized using principal component analysis which reduced the multiple (high-dimensional) features to just 2 dimensions, enabling the clusters to be plotted for visual inspection and verification. The resulting cluster labels were then mapped back onto the original feature data set to allow each cluster to be analyzed. Independent *t* tests were carried out to evaluate the significance of the difference in the means of the groups.

**Table 1:** Features used for k-means clustering.

Feature	Description
Unique days	This is the number of unique days the user accessed the chatbot.
Tenure	This is the number of days between the first use and last use of the chatbot.
Mood logs completed	This is the number of times the user recorded their mood over their period of use of the app. This gives an indication of how many times the app was used as the user’s mood was requested each time they used the chatbot, there may be multiple mood logs for each day.
Conversations accessed	This is the number of conversations accessed during their period of use of the chatbot.
Total interactions	This is the total number of interactions with the chatbot.

### Feature Usage Analysis

The key feature of the chatbot, a series of scripted “conversations,” was chosen for further analysis. Users could access these conversations via the main menu and were asked to rate the conversation as “good,” “neutral,” or “bad” on completion. The log data was analyzed to examine the number of times each conversation was accessed during the prepost study period, the percentage of users that accessed each conversation, and the rating awarded by users on the completion of each conversation. The date and time when each user accessed any conversation were logged, making it possible to create a daily-ordered set of conversations for each user for analysis. From these sets, it was possible to examine associations between conversations; in other words, examine the pathway from one conversation (the antecedent) to the next (the consequent) using association rule mining.

## Results

### User Interactions

There were a total of 1403 individual users that accessed the app between January 24, 2022, and June 22, 2022. Only data from users older than 18 years were included in the analysis; thus, the results report on data from 579 adult users, of whom 348 (60.1%) were recruited specifically for a 12-week prepost study [25]. The majority of users identified as female (387/579, 66.8%), male (153/579, 26.4%), or other (6/579, 1%), while other users preferred not to say (12/579, 2.1%) or elected not to answer (21/579, 3.6%). Nonresponses were treated as unknown. The ages of participants were well distributed, with responses of 18-24 (190/579, 32.8%), 25-34 (150/579, 25.9%), 35-44 (95/579, 16.4%), 45-54 (77/579, 13.3%), 55-64 (56/579, 9.7%), and >65 (11/579, 1.9%).

**Figure 3:**
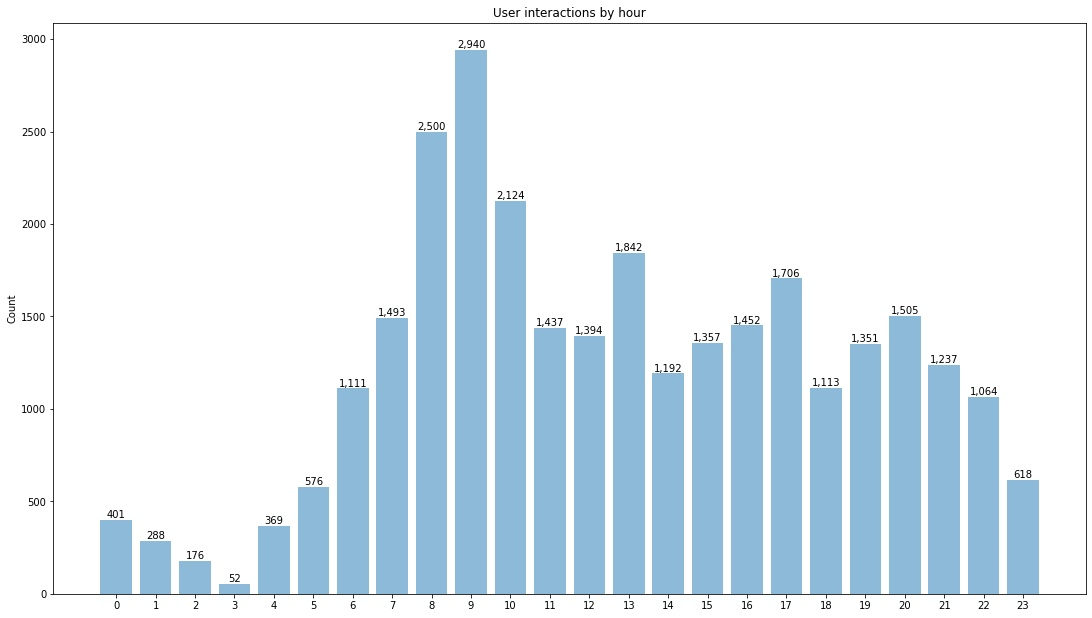
User interactions over the course of the day.

Over the study period, from January 24, 2022, to June 24, 2022, there were a total of 29,298 user interactions with the app, averaging 246 interactions per day. While users interacted with the app throughout the day, peaks in interactions can be seen at key times of the day: breakfast (8 AM-10 AM), lunch (1 PM), and the end of the working day (5 PM) (Figure 3).

**Figure 4:**
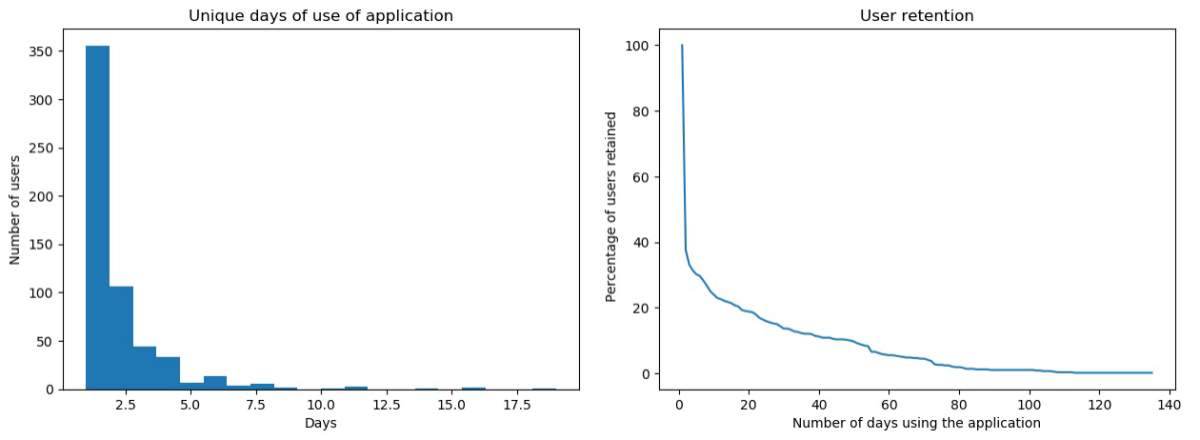
Unique days of use of the chatbot (left) and user retention curve (right).

A large proportion of users interacted with the app for a short period of time, with 440 users (76%) interacting for less than 10 days. Analysis of the number of unique days of interaction with the app also showed that although there were users who interacted with the app over almost the whole study period, these interactions were sporadic, with the most ardent user interacting with the chatbot over 19 unique days (Figure 4). Overall user retention shows a steady drop-off in users over time (Figure 4). The average tenure of a user was 11.4 days.

A total of 6 features representing the behavioral usage of the app were selected for k-means clustering to identify the different types of users accessing the app. These features represent user characteristics, including the number of unique days the user accessed the app, the tenure of each user, the number of mood logs recorded, the number of conversations accessed, and the total number of interactions with the app.

**Figure 5:**
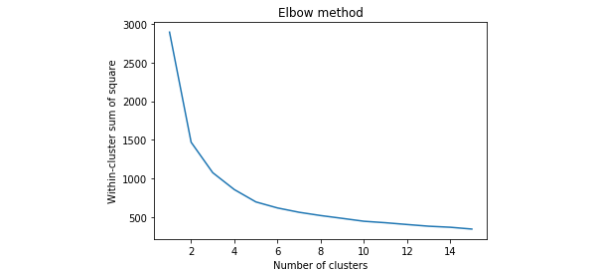
Elbow plot showing the optimum number of clusters.

The optimum number of types of users (clusters) was determined using the elbow method. This method runs k-means clustering on the data set for a range of k values (eg, k=1-15), calculating the average distances to the centroid from all data points, known as the within-cluster sum of squares. These distances are then plotted on a graph, which will show where the distances fall, creating an elbow in the graph. This elbow represents the optimum k value for the clustering solution. Figure 5 shows the results, indicating 2 possible solutions, k=2 and k=3 in this case.

### User Types

**Figure 6:**
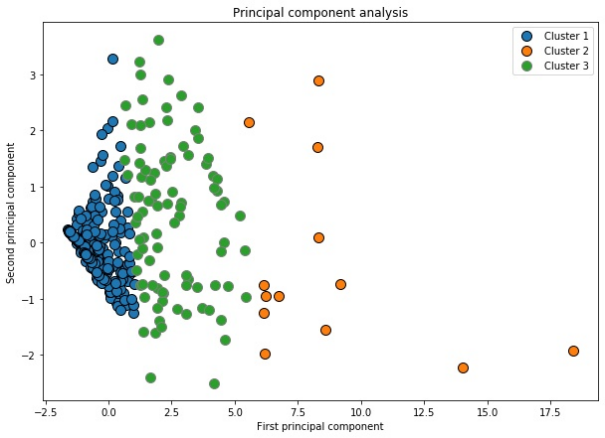
Principal component analysis plot of output from the k-means algorithm (3-cluster solution).

Both a 2- and 3-cluster solution were explored. Using k=3 results in a similar principal component analysis plot as in the 2-cluster solution, with 3 well-defined clusters with distinct usage characteristics (Figure 6).

**Figure 7:**
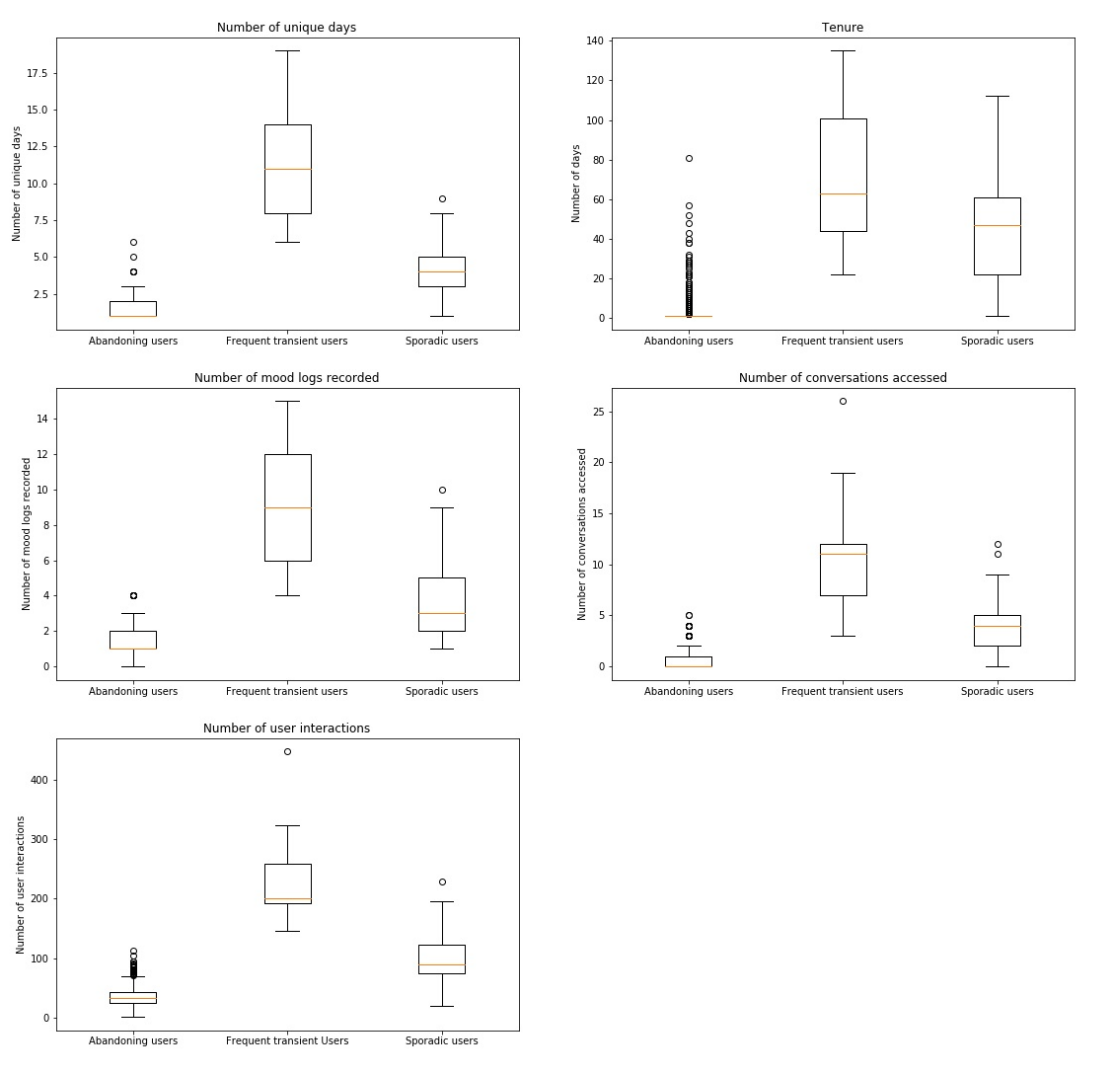
Boxplots of feature values for the different clusters (3-cluster solution).

The 3-cluster solution (Figure 7) appeared to be a refinement of the 2-cluster solution, with the largest cluster equating to “abandoning users” and the remaining 2 clusters revealing a more granular look at more invested users. Analysis of the archetypal characteristics of these 3 clusters (Table 2) suggested that “abandoning users” (473/579, 81.7%), “frequent transient users” (13/579, 2.2%), and “sporadic users” (93/579, 16.1%) would be appropriate labels.

Abandoning users generally access the app on 1 or 2 unique days, with an average tenure of 3.6 days (Table 2). They recorded lower numbers of mood logs (1-2), accessed fewer conversations (0-1), and had fewer interactions with the chatbot (25-43) compared to the other 2 clusters. At the other extreme, frequent transient users accessed the app between 8 and 14 unique days, with an average tenure of 68.9 days (Table 2). They recorded the highest number of mood logs (6-12) and conversations (7-12) and had the highest number of interactions with the app (193-258). While sporadic users only used the app for a small number of unique days (3-5), they did so over a longer period (22-61 days) (Table 2). In all other metrics, they exceeded those recorded by abandoning users but did not achieve the numbers attributed to frequent transient users: mood logs (2-5), conversations (2-5), and interactions (20-123). Independent *t* tests on these results found that the 3 archetypes are significantly different statistically across all metrics (*P*<.001).

**Figure 8:**
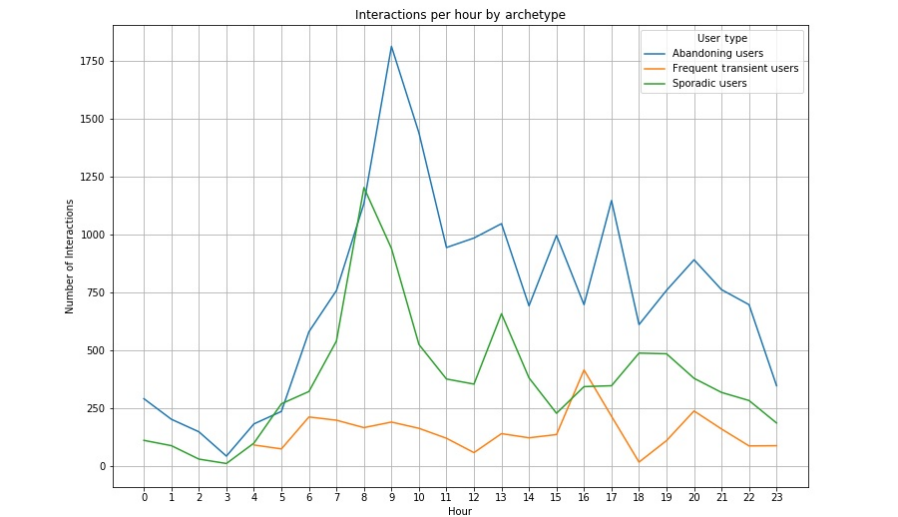
Average number of interactions per user each hour by archetype.

Daily patterns of usage of ChatPal differed for each archetype (Figure 8). Abandoning users recorded low numbers of interactions with the app over the course of the day, with a small peak of usage in the morning (9 AM). Interactions for sporadic users were higher than those seen for abandoning users, and in general, lower than those for frequent transient users, the exception being in the morning (8 AM), when interactions for sporadic and frequent transient users were the same. Sporadic users showed peak usage times around breakfast (8 AM) and lunch (1 PM). Frequent transient users generally recorded the highest number of interactions with the app over the course of the day, with frequent peaks in usage. These users showed high levels of interactions over most of the morning (4-9 AM), with further peaks in usage at lunchtime (1 PM), afternoon (4 PM), and evening (8 PM).

**Table 2:** Archetypal characteristics for each cluster (3-cluster solution).

Feature	Cluster 1: abandoning users	Cluster 2: frequent transient users	Cluster 3: sporadic users
Users, n (%)	473 (81.7)	13 (2.2)	93 (16.1)
Unique days, mean (SD)	1.3 (0.7)	11.2 (3.9)	4.0 (1.7)
Tenure, mean (SD)	3.6 (8.1)	68.9 (35.4)	43.0 (25.2)
Mood logs completed, mean (SD)	1.3 (0.8)	8.8 (3.8)	3.8 (1.9)
Conversations accessed, mean (SD)	0.8 (1.0)	10.8 (6.3)	4.0 (2.4)
Total interactions, mean (SD)	36.7 (16.8)	229.3 (83.0)	96.1 (36.6)

### Feature Usage Analysis

**Figure 9:**
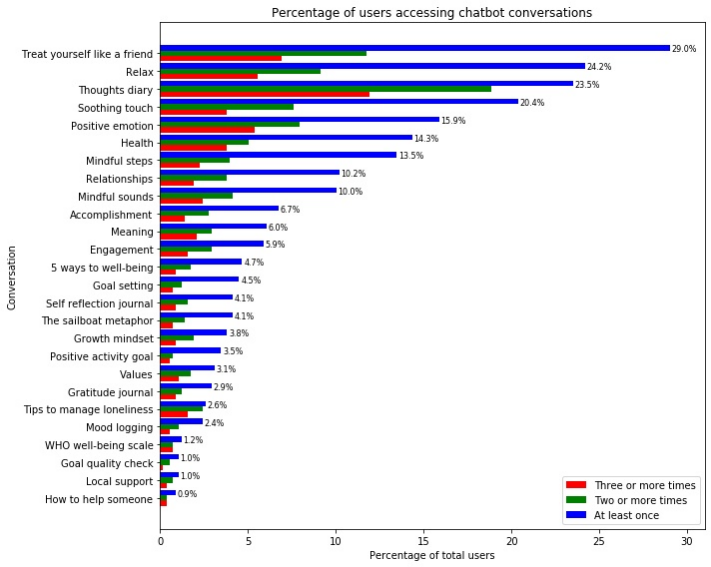
Percentage of users accessing chatbot conversations. WHO: World Health Organization.

**Figure 10:**
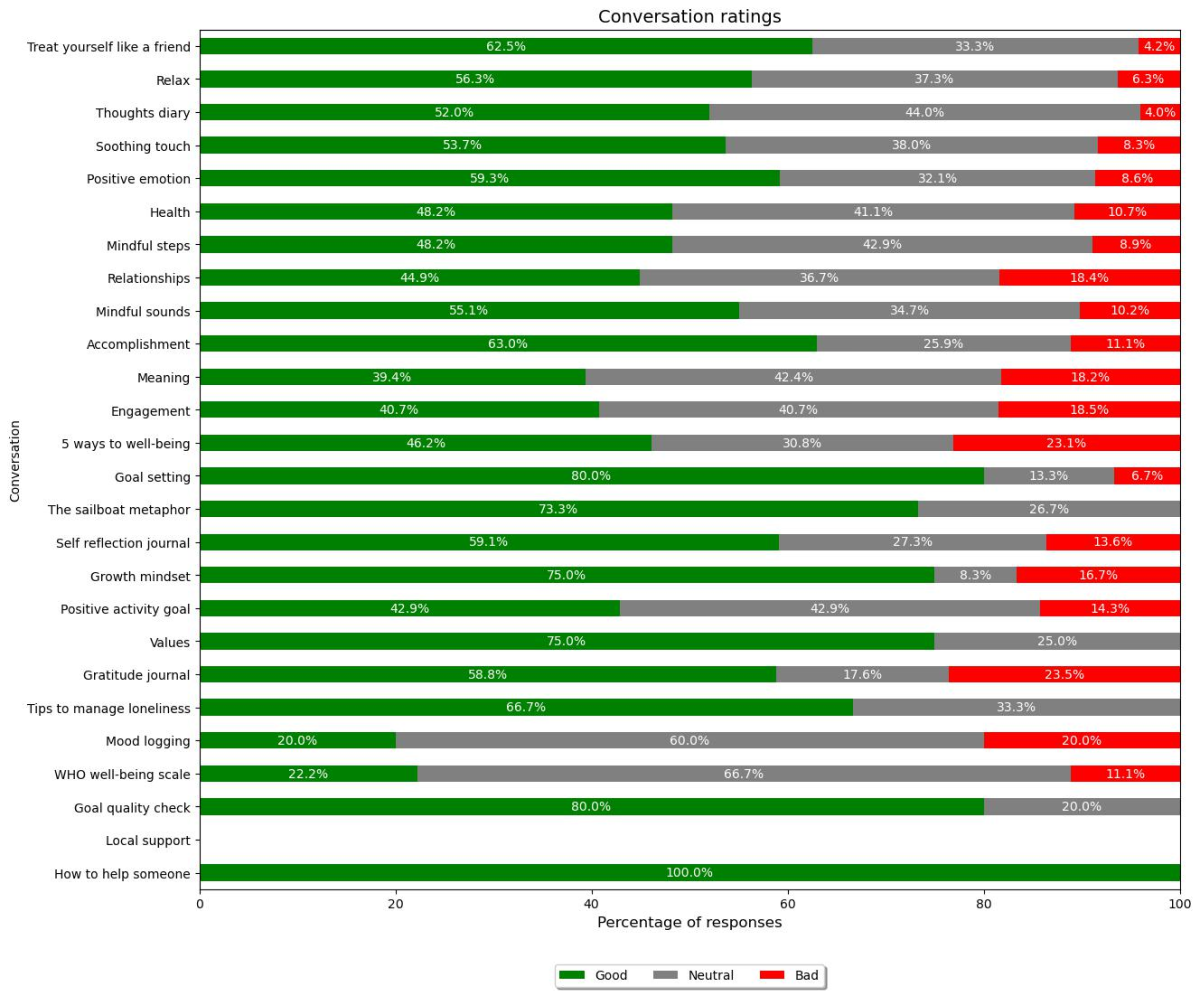
Conversation ratings. WHO: World Health Organization.

A key element of the app was the provision of mental well-being conversations between the chatbot and the user. Analysis of users’ choice of conversations showed that of the 579 users, almost one-third chose to access the “Treat yourself as a friend” conversation (168/579, 29%), with over one-fifth of users accessing the “Relax (140/579, 24.2%),” “Thoughts Diary” (136/579, 23.5%), and “Soothing Touch” (118/579, 20.4%) conversations. Of these, only 68 (11.7%) returned to the “Treat yourself as a friend” conversation on 2 or more occasions, with this number falling to 40 (6.9%) returning on 3 or more occasions. The conversation with the highest return rate was “Thoughts Diary” (109/579, 18.8% for 2 or more occasions; 69/579, 11.9% for 3 or more occasions), while the conversation with the lowest return rate was “How to Help Someone” (2/579, 0.3% for both metrics) (Figure 9). The top conversations received good ratings, reflecting their popularity with users. “Treat yourself as a friend” received the highest percentage of good ratings of the 4 (62.5%), followed by “Relax” (56.3%), “Thoughts Diary” (52%), and “Soothing Touch” (53.7%). Interestingly, the conversations that were used the fewest number of times elicited more positive ratings. “How to help someone,” which was only accessed by 0.9% of the total users, resulted in a 100% good rating, with “Goal Setting” and “Goal Quality Check” receiving 80% good ratings (Figure 10).

Analysis of the number of times each user accessed conversations showed that the majority of users used each conversation between 1 and 3 times. “Thoughts diary,” “tips to manage loneliness,” and “WHO well-being scale” had a median number of users of 3. A small number of users who accessed these conversations multiple times are represented as outliers on the graph (Multimedia Appendix 2).

**Figure 11:**
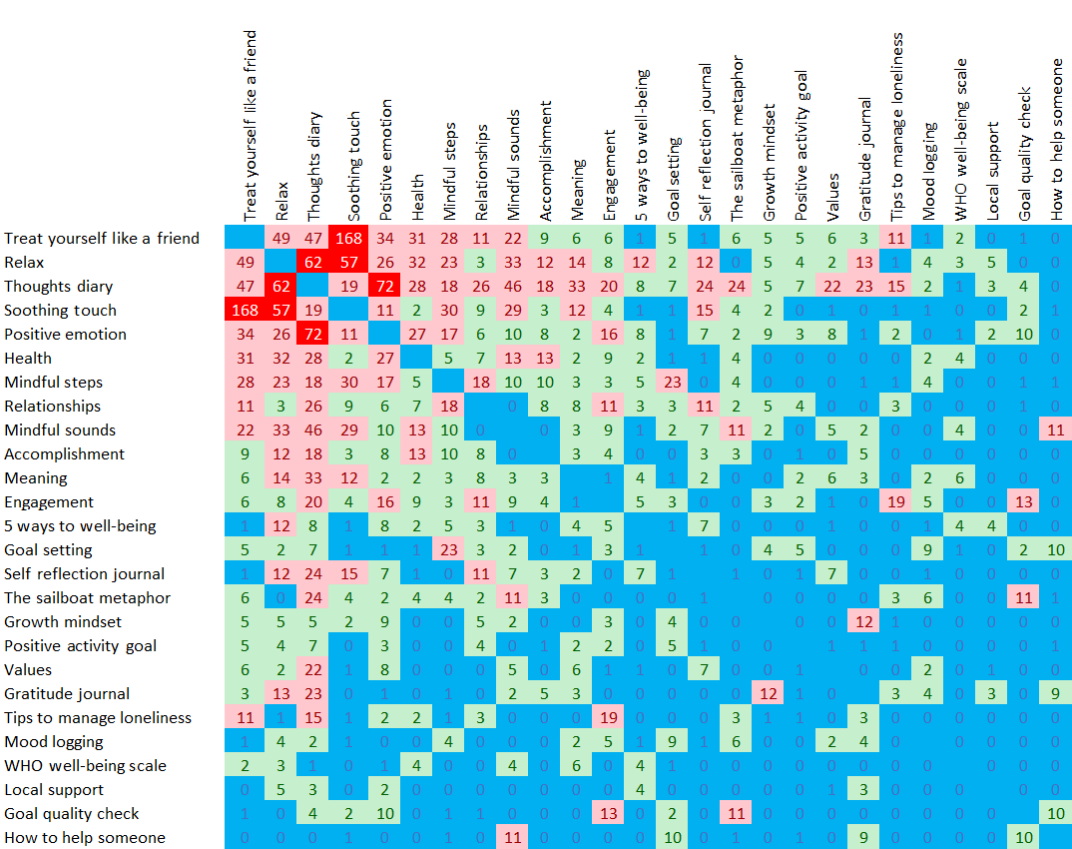
Associations between conversations (blue=low <2, green=average 2-11, pink=high 12-50, red=very high >50). WHO: World Health Organization.

The log data relating to “conversations” were examined for each user on a daily basis in order to provide insight into how users accessed conversations. Each time a user moved between 2 conversations, no matter the direction, the transition was counted. Statistical analysis of the resulting 307 transitions revealed the median to be 4, the lower quartile to be 2, and the upper quartile to be 11. Using these statistics, Figure 11 shows the results of the analysis, with low numbers of transitions represented in blue (<2), average represented in green (2-11), high in pink (12-50), and very high in red (>50). The transition between “treat yourself as a friend” and “soothing touch” was the most popular, being performed 168 times. Additionally, high on the scale were the transitions between “positive emotion” or “thoughts diary” (72) and “relax” or “thoughts diary” (62). Association rule mining supports these findings, with the linkage between “treat yourself as a friend,” and “soothing touch” receiving the highest support of 0.16 and the highest support of a rule with more than one antecedent being “soothing touch” or “thoughts diary” with “treat yourself like a friend” with support of 0.072 (Table 3).

**Table 3:** Summary of results of association rule mining on conversations ordered by lift.

Antecedents	Consequents	Support	Confidence	Lift
Soothing touch	Treat yourself like a friend	0.160	0.712	2.069
Treat yourself like a friend	Soothing touch	0.160	0.463	2.069
Thoughts diary	Treat yourself like a friend	0.121	0.391	1.135
Treat yourself like a friend	Thoughts diary	0.121	0.352	1.135
Relax	Treat yourself like a friend	0.104	0.396	1.150
Treat yourself like a friend	Relax	0.104	0.302	1.150
Soothing touch	Thoughts diary	0.094	0.420	1.352
Thoughts diary	Soothing touch	0.094	0.303	1.352
Positive emotion	Thoughts diary	0.093	0.475	1.530
Thoughts diary	Positive emotion	0.093	0.299	1.530
Soothing touch	Relax	0.092	0.410	1.562
Relax	Soothing touch	0.092	0.350	1.562
Relax	Thoughts diary	0.090	0.342	1.101
Thoughts diary	Relax	0.090	0.289	1.101
Positive emotion	Treat yourself like a friend	0.089	0.453	1.314
Treat yourself like a friend	Positive emotion	0.089	0.257	1.314
Health	Thoughts diary	0.073	0.370	1.193
Thoughts diary	Health	0.073	0.236	1.193
Soothing touch, thoughts diary	Treat yourself like a friend	0.072	0.767	2.229
Treat yourself like a friend, thoughts diary	Soothing touch	0.072	0.595	2.654
Soothing touch, treat yourself like a friend	Thoughts diary	0.072	0.452	1.456
Soothing touch	Treat yourself like a friend thoughts diary	0.072	0.322	2.654
Thoughts diary	Soothing touch, treat yourself like a friend	0.072	0.232	1.456
Treat yourself like a friend	Soothing touch, thoughts diary	0.072	0.210	2.229
Mindful sounds	Thoughts diary	0.071	0.512	1.649
Thoughts diary	Mindful sounds	0.071	0.229	1.649

## Discussion

### Principal Results

The majority of users (348/579, 60%) of the ChatPal chatbot app were recruited as volunteers in a 12-week prepost study [25]. User interactions occurred at all hours of the day, with the majority being during working hours. It was notable that spikes in usage occurred at the start of the working day (9 AM), lunchtime (1 PM), and end of the working day (5 PM). This may simply reflect the fact that users felt they could devote some time to evaluating the app at these times, or it could reflect a need for support during times of the day when users are not pressured by other commitments. User interactions with the app late into the evening and through the night may indicate the need for support at these times. Recent studies found that the COVID-19 pandemic resulted in increased sleep disturbances and worsening mental health in the general population [26-28]. This is one advantage of offering digital solutions in mental health care, as these technologies can be accessed 24/7 when traditional face-to-face services are unavailable.

Analysis of user interaction with the chatbot conversations reflects a high proportion of “abandoning users.” The majority of conversations were accessed only once, and only a small percentage of total users accessed conversations more than once. Interestingly, the conversation with the highest return rate was the “thoughts diary,” where users could record their thoughts and feelings and review them in subsequent sessions. This indicates that these users felt comfortable enough with the app to share their feelings, and they presumably felt some benefit from doing so as they returned on multiple occasions. Journaling about stressful events has been shown to be beneficial for individuals to understand and make sense of what happened [29].

The majority of conversations received a “good” or “neutral” rating, with “how to help someone” receiving a 100% good rating. While this is encouraging, it is important to recognize that the “how to help someone” conversation received the lowest percentage of overall users. Given these low figures, they may be biased by a small number of returning users. It is interesting that “how to help someone” was the least accessed conversation, as it suggests that people are not using the app to access information that would help others but more for resources to benefit their own well-being.

Analyzing users’ transition from one conversation to another revealed that the most frequent transition was the reciprocal journey from “treat yourself like a friend” to “Soothing Touch,” followed by transitions between the “positive emotion” or “thoughts diary” and “relax” or “thoughts diary” pairs of conversations. Association rule mining supported these findings as these 3 conversations, “soothing touch,” “thoughts diary,” and “treat yourself like a friend,” were linked, with “thoughts diary” having strong links between the other 2 in either direction. This seems understandable as “treat yourself like a friend” and “positive emotions” are both conversations in which the user is writing something and may want to see it in the “thoughts diary” (saving the written message to the “thoughts diary” is suggested at the end of the “treat yourself like a friend” script, which may prompt the user to go there next). Additionally, “treat yourself like a friend,” “soothing touch,” and “relax” are all short dialogues or exercises, so users may want to go on chatting and access further conversations. This also demonstrates the value of incorporating efficient scripts into the app, providing quick exercises for users needing support [30].

Further analysis of the chatbot menu structure would provide clarity on whether these conversations naturally complement each other or whether some bias has been introduced. In either case, the associations observed will provide valuable data for the further development of the app, not only in highlighting the conversations that fit naturally with each other and presumably provide the most support for users, but also in highlighting those that do not.

### Comparison With Prior Work

The majority of users (76%) accessed the app for a period of less than 10 days with 62.7% accessing ChatPal for 1 day. While the remaining 24% accessed the app for more than a 10-day period, analysis of the number of unique days of usage shows that the app was used for a maximum of 19 unique days by an individual user with the average being 2 unique days. While the app was designed to be beneficial to all users’ mental well-being, the results indicate that only a small proportion of participants became invested in using the app. While this lack of user retention is disappointing, it is not unusual. Benchmarking the ChatPal app against a study of 93 mobile apps, 59 of which specialized in mental health [31] shows that ChatPal performed better than average with a drop-off rate in the first 10 days of 77% compared with the average of 80% and a drop off between day 15 and day 30 of 7.1% compared with an average of 20% for other mental health apps. While this indicates that ChatPal has a better than average retention of users over the first 10 days, it also suggests that the remaining users may find benefit in using the app leading to a low drop off of users between days 15 and 30. A recent study [32] found that digital interventions for depression with human guidance yielded better results compared to digital interventions without any external guidance. Perhaps if chatbots such as ChatPal were used in conjunction with health professionals this may encourage usage and result in improved benefits to users, however further work would be needed to confirm this.

Discovering the types of users accessing chatbots and their patterns of usage is essential for the further development and targeting of the services provided by the app. K-means clustering was used to discover 3 different groups of users (abandoning users, frequent transient users, and sporadic users) based on 6 key features extracted from the log data. Further analysis found significant differences between the features for each group of app users.

Abandoning users, making up 87.2% of total users, generally accessed the app on 1 or 2 unique days with an equally low average tenure of 5.7 days. They had significantly less interactions with the app resulting in less moods being logged than invested users and participated in significantly less conversations. In contrast, frequent transient users and sporadic users were more generally invested users, accessing across more unique days with an average tenure of 50.5 days and 68.9 days, respectively. During this time, these users logged their mood on more occasions and generally interacted with the chatbot a lot more, accessing between 5 to 10 different conversations. This gives rise to the possibility that a small number of users found value in the support offered by the chatbot. These results are comparable with previous analyses of the initial trial of the prototype chatbot [16]. Further analysis is needed to explore the demographics of the users in each of these archetypes in order to understand why users belong to the archetype they are associated with and provide insight into users who repeatedly use specific features in order to understand if certain subgroups of users tend to favor certain features over others. The AI could then be used to direct users toward these features within the bot. This may also have the added benefit of increasing adherence and retention.

### Policy and Practice Implications

We have included policy and practice implications based on the findings from this study, which may benefit others who are designing digital mental health technologies (Textbox 1).

**Recommendations for policy and practice.**

- As the app was used by some during the night, conversations or exercises specifically for treating insomnia could be added to the available conversations and offered when users access the app during the nighttime. However, promoting screen time during the evening or middle of the night may not be appropriate.
- Given the drop-off rate of users over time, strategies could be used to improve user retention. For example, further development of the chatbot, to make it more spontaneous, “remembering” the last conversation or mood, more personalization and added daily reminders.
- Three main groups of app users were identified: “abandoning users,” “sporadic users,” and “frequent transient users.” While there will always be abandoning users due to the free nature of the app, the chatbot could have other engaging features such as peer communication and support to encourage use.
- The most used conversations within the chatbot were not necessarily the best-liked. The app could adapt based on user feedback, possibly changing the order of conversations offered based on ratings or allowing users access to the ratings in order to make informed decisions.
- Users’ moods were only collected at the start of each session. In order to gauge the effectiveness of the app, user moods could be collected at the start and end of each session.
- The World Health Organization-Five Well-being Index (WHO-5) well-being scale within the chatbot gave no feedback to the user. It may be more beneficial to display the score as a time series graph and provide insights to the user on their well-being over time.

### Limitations

Despite the detailed event log data captured, tracking the length of individual sessions was difficult due to the lack of “end of session” variable. Almost all users chose not to select the “need to go” option in the chatbot which would have been recorded as “end of session,” and instead must have exited or closed the app which was not recorded in the logs. For this reason, user sessions were tracked on a daily basis. An additional variable to track the end of the session would be useful to explore individual user sessions rather than sessions per day. This omission may also have contributed to the low retention rates as the app was unable to provide little feedback to the user. With the addition of session data, user moods could be logged giving the app the ability to personalize its interactions with the user. For example, returning users could be asked “are you still feeling…” or “Hello again”. In addition, if users’ moods were asked at the start and end of each session, it would be possible to gauge changes in well-being that resulted from using the app. A bias may also have been introduced into the way users interacted with the app due to the rigid structure of the menus presented to the user. These may have caused some users to follow the list of available conversations in the order they are presented in the first instance before deciding which conversations provided the most support for their circumstances. Mood logs were asked each time the app was launched and, while these were used in the discovery of user archetypes, no further analysis was possible due to the following reasons. As users were only asked about their mood at the start of the session, there is nothing to compare the responses to. The addition of session data and requesting a user’s mood at the end of a session would have allowed comparisons to be made and facilitated the analysis of the effect of the app on the users’ mental well-being.

The app recorded interactions based on server time, not local time. This means that log data will be incorrect by 1 hour for Swedish users and 2 hours for Finish users. Not all users reported their country, thus no adjustments were made to account for differing geographical locations. As the WHO-5 scale questions were optional, there was no substantial data collected during the trial period.

### Conclusions

The ChatPal app was developed as part of a research project into the use of chatbots to promote positive mental health and well-being. From the log data gathered, 3 main types of users accessed the chatbot: abandoning users, sporadic users, and frequent transient users. Due to the high numbers of abandoning users, it is difficult to evaluate the effectiveness of the app though analysis of the other 2 groups of invested users allows a glimpse into the benefits the app could bring to a user’s mental well-being. It is clear that some users returned to the app on several occasions although there is no evidence that this was linked to improvement in well-being. Improvements incorporated in future versions of the app suggested in this paper would provide data on participants’ moods at the beginning and end of each session and would provide evidence as to what effect the app has on users. Analysis of user transitions from conversation to conversation indicated that some dialogues may complement each other and provide targeted support to users although further analysis may be necessary. Future versions of the chatbot should be enhanced to make each interaction with the user more personalized, learning and adapting from the previous interactions with each user.

## Appendix

Multimedia Appendix 1 Overview of available content in the ChatPal chatbot.

Multimedia Appendix 2 Conversations by number of times accessed.

## Abbreviations

EMA: ecological momentary assessment
WHO-5: World Health Organization—Five Well-being Index
