# Supplementary figures and images for: A Mental Health and Well-Being Chatbot: User Event Log Analysis
Source: JMIR Mhealth Uhealth. 2023 Jul 6;11:e43052. doi: 10.2196/43052 (PMC10360018; doi:10.2196/43052)

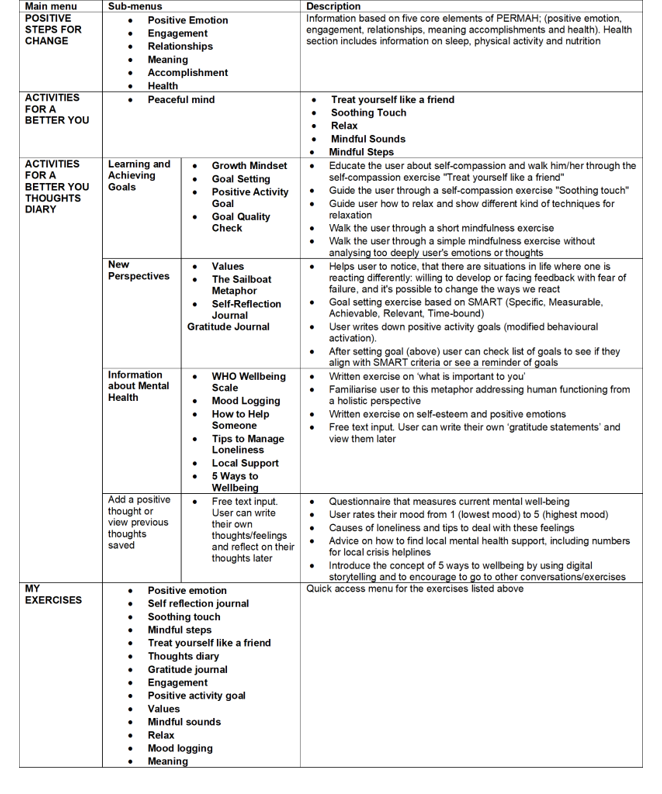

Supplement: Multimedia Appendix 1 [file mhealth_v11i1e43052_app1.png]

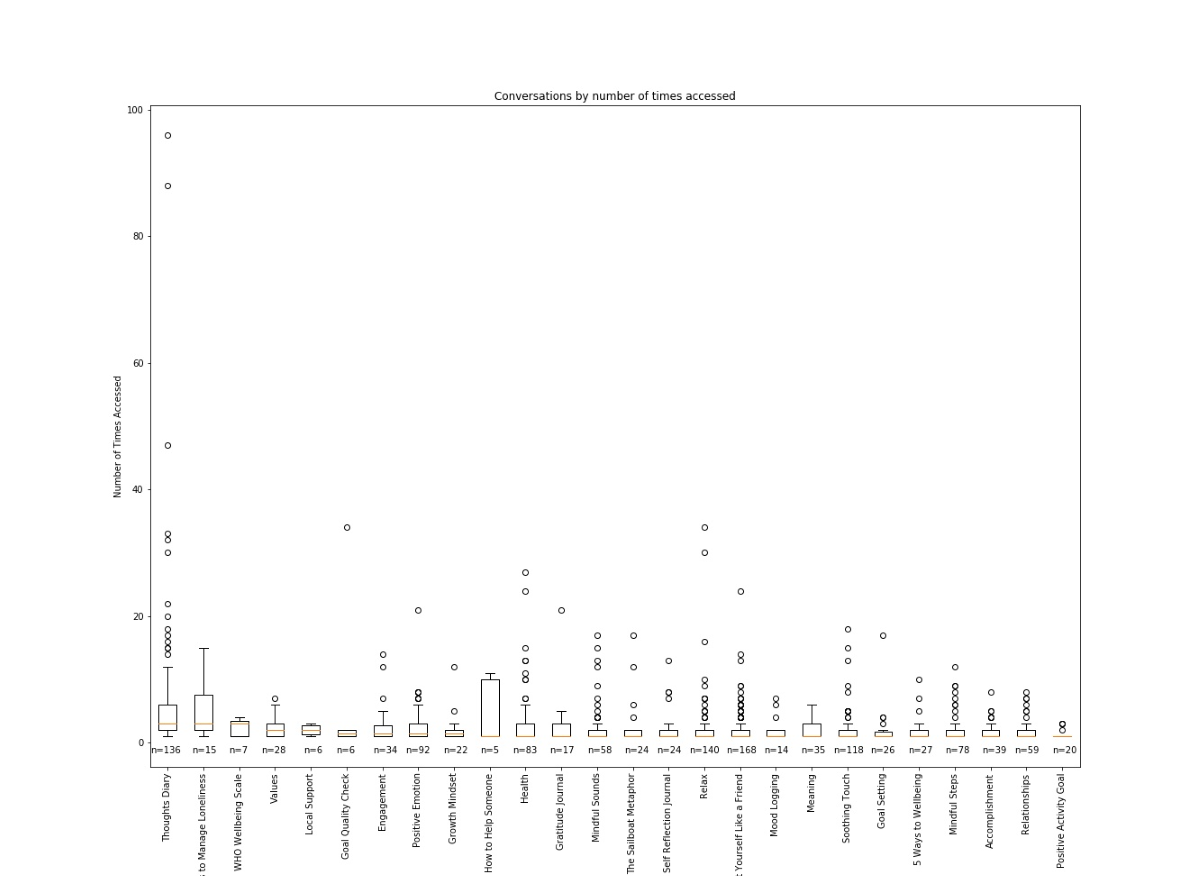

Supplement: Multimedia Appendix 2 [file mhealth_v11i1e43052_app2.png]
